# Development and Validation of the Age Integration Scale: Measuring Older Adults’ Intergenerational Attitudes

**DOI:** 10.1093/geroni/igaa057.1451

**Published:** 2020-12-16

**Authors:** Carly Roman, Elizabeth Zelinski, Christopher Beam

**Affiliations:** 1 University of Southern California, Los Angeles, California, United States; 2 Dornsife College of Arts and Letters, Los Angeles, California, United States

## Abstract

Opportunities for age integration (i.e., more intergenerational connections) are increasing among older adults, as a greater number live longer and are capable of interacting with younger individuals. Attitudes toward other generations can be considered a barrier that promotes or discourages intergenerational connections. While existing measures tend to focus on attitudes toward aging or older adults, they do not evaluate older adults’ attitudes toward younger individuals. Item response theory (IRT) was used to create an attitude scale evaluating older adults’ perceptions of their own age integration with younger individuals. Convenience sampling was used to recruit 100 older adults (55+ years old) who completed a 30-item age integration survey assessing their agreement (on a 5-point Likert scale) with statements about intergenerational beliefs and intentions to have intergenerational connections. IRT analyses supported a 10-item age integration scale indicating a unidimensional construct. The scale consists of items with moderate discrimination and difficulty levels on a 4-point Likert scale. Composite reliability of the 10-item scale was 0.83, which is considered substantial. Tests of convergent validity demonstrated that the total scale score correlated 0.68 with generativity. Discriminant validity tests suggest that the scale does not correlate strongly with satisfaction with life, purpose, depression, need for cognition, or considerations for future consequences, as the correlations ranged from -0.01 to 0.38. This novel measure provides an important, less-considered perspective of intergenerational relationships by assessing older adults’ attitudes toward younger individuals. Future studies will validate this scale in a larger, more generalizable sample.

